# Implementation of strategies to promote men’s health-seeking behaviour in a rural village of Limpopo Province, South Africa: a study protocol

**DOI:** 10.3389/fpubh.2026.1874114

**Published:** 2026-08-24

**Authors:** Lazarros Chavalala, Lufuno Makhado

**Affiliations:** Department of Public Health, University of Venda, Thohoyandou, South Africa

**Keywords:** health care, health seeking, implemenation, improve, men, strategies

## Abstract

**Background:**

Men often experience challenges in seeking healthcare when they become ill or develop conditions requiring medical attention. They frequently seek care as a last resort and, in some cases, do not seek care at all, even when their health deteriorates.

**Aim:**

This study aims to implement and evaluate strategies designed to promote men’s health-seeking behaviour in selected rural areas of Limpopo Province.

**Methods:**

The Participatory Action Research approach will be employed to guide the implementation of the strategies. The stakeholder committee members will be purposively selected from relevant stakeholders and community members. Total population sampling will be used to include all eligible men attending community dialogues and workshops as participants and beneficiaries. Focus group discussions, key informant interviews and evaluation questionnaires will be used to collect data. Qualitative data will be analysed thematically, while quantitative data will be analysed using both descriptive and inferential statistics.

**Conclusion:**

The findings will be used to measure the effectiveness of the strategies and to identify gaps for further refinement.

## 1 Introduction

In many parts of the world, men engage with health services less frequently than women, are less likely to access preventive services, and are more likely to drop out of care (1). Men often delay seeking help for health-related problems and consider consulting only when medical conditions become severe and potentially difficult to treat (2). Given that men underutilise health services, numerous studies, including those conducted by (3–6), explore factors contributing to men’s poor health-seeking behaviour. Following studies that identified factors contributing to men’s poor health-seeking behaviour, strategies were recommended based on the identified factors. The recommended strategies included patient-physician communication strategies, community-based interventions, the establishment of male clinics, peer education programmes, and the introduction of men’s sheds (7–11). However, gaps were identified that contributed to the ineffectiveness of some of the developed strategies, including the absence of practical approaches to encourage men who avoid using health services to engage with them. Given the identified gaps and factors, the current authors conducted a study to uncover contemporary factors contributing to men’s poor health-seeking behaviour in rural areas. The study’s findings were then used to develop 14 strategies that can be employed to improve men’s health-seeking behaviour in rural areas. The strategies were then validated through expert and stakeholder consultations using the Chinn and Kramer model (12). However, these strategies have not yet been implemented and evaluated in a real-world setting. Therefore, this study aims to implement the developed strategies to improve men’s health-seeking behaviour in rural areas. The study will assist in evaluating the effectiveness of the proposed strategies and aspects that may need to be revised to improve men’s health-seeking behaviour. Moreover, the implementation of the developed strategies also supports the achievement of Sustainable Development Goal (SDG) 3, which seeks to achieve good health and well-being for all, including men. This study is guided by the Capability, Opportunity, Motivation–Behaviour (COM-B) model. The model states that for an individual to change their behaviour, there must be capacity, opportunity and motivation (13). In this study, capability refers to men’s knowledge of health conditions, their statuses, and the benefits of using healthcare services. Opportunity includes the availability of free healthcare services at public health facilities, including support from community leaders, healthcare providers, and family members. Motivation refers to beliefs, attitudes, intentions, and social norms that influence men’s decisions to seek healthcare.

The proposed intervention strategies are designed to improve capability through health education, enhance opportunity through stakeholder engagement and community mobilisation, and strengthen motivation through community dialogues aimed at challenging harmful social norms and promoting positive health behaviours. This protocol describes the procedures that will be followed to implement and evaluate the proposed strategies.

## 2 Aim and objectives

The overall aim of this study is to implement and evaluate the effectiveness of developed strategies to improve men’s health-seeking behaviour in selected rural areas of Limpopo, through the following objectives:

- Establish working committees consisting of the Department of Health PHC directorate, facility managers, professional nurses, community leaders, traditional healers, representatives of men living in the community, religious leaders, and NGOs.
- Engage the Department of Health management, health care workers, men, traditional healers, and community leaders in the adoption of developed strategies.
- Collaborate with stakeholders in the implementation of strategies
- Train stakeholders on developed strategies and implementation
- Evaluate the effectiveness of these strategies through iterative cycles of reflection and action (see Table 1).

**Table 1:** Strategies to improve men’s health-seeking behaviour in Limpopo Province, South Africa.

Strategy	Purpose
1. Implementation of the best employee recognition programme for employees serving males in health facilities	Encourage employees to engage with male patients respectfully and appropriately.
2. Implementation of male patients’ satisfaction surveys	Determine male patients’ satisfaction level, and identify concerns and areas that need to be improved in the provision of health care services.
3. Intensify compliance with policies and guidelines applicable to health service provision and ethical conduct.	Ensure compliance with policies and guidelines governing health services provision and ethical conduct of health professionals towards male patients.
4. Reduction of waiting period for males in health facilities	Reduce the time male patients spend in the health facility.
5. Regular staff training	Train health workers to communicate effectively with male patients Encourage health workers to communicate the reasons and factors contributing to delays in providing patients with services in the health facility. Remind health workers of guidelines and policies applicable in providing health services and interacting with patients, including males. Encourage health workers to adhere to ethical guidelines Ensure compliance with policies and guidelines governing health services provision and ethical conduct
6. Employ male nurses and community health workers (CWHs)	To accommodate men who visit public health facilities and feel uncomfortable being assisted by female clinicians. Use CWHs to encourage men to utilise health services and treatment adherence
7. Use available technology to influence men’s health-seeking behaviour	Use social media platforms such as Facebook, X (previously known as Twitter), and Podcasts to educate men about the importance of utilising health services and regular disease screening. Promote services offered at public health facilities tailored for men and the benefits of the services. Create adverts that display the benefits of utilising health services to encourage men to utilise health services. Provide remote access to health services using e-health platforms such as telemedicine.
8. Collaborate with community leaders, Faith-Based Organisations (FBOs), and Community-Based Organisations (CBOs)	Create a working relationship with community leaders, Faith-Based Organisations (FBOs), and Community-Based Organisations (CBOs).
9. Collaborate with traditional healers	Establish working relationships with traditional healers serving males
10. Implement community outreach campaigns targeting males	Change men’s negative attitude towards public health services Educate males about how masculine beliefs and control over one’s health affect individual health. Educate the community, including men, about service packages offered in health facilities to reduce the stigma attached to using health services. Cultural practices and traditional healing in dealing with diseases
11. Advocacy for employed males to access health services	Advocate for employed males to be given time off and allowed to access health services.
12. Proper monitoring of the implementation of the South African National Integrated Men’s Health Strategy 2020–2025	Ensure proper implementation of the South African National Integrated Men’s Health Strategy 2020–2025
13. Introduction of male-dedicated sections in existing health facilities	Enable male patients who do not feel comfortable being assisted by female clinicians to be assisted by male clinicians.

## 3 Methods

### 3.1 Study design

**Figure 1:**
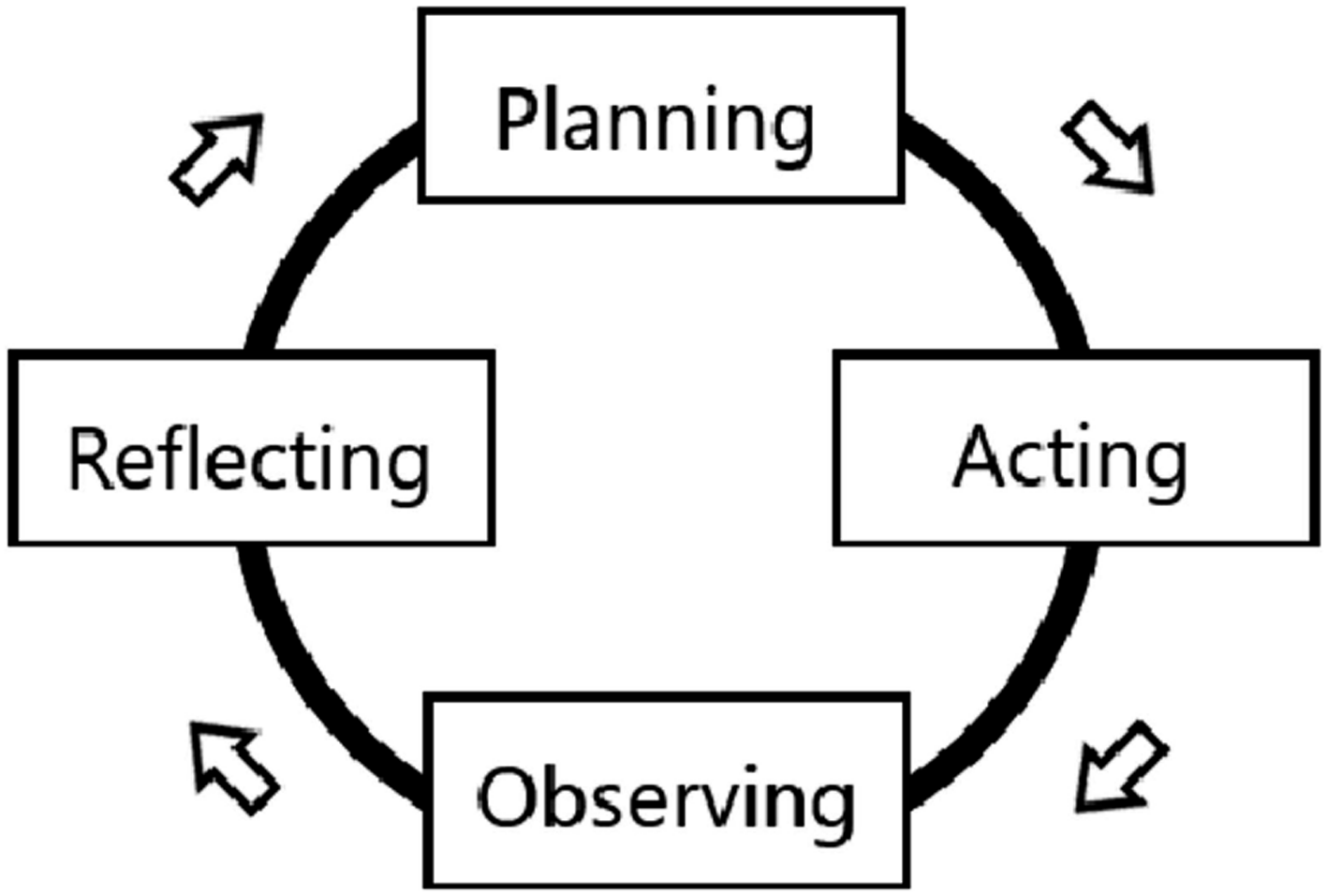
Participatory action research cycles.

Participatory Action Research (PAR), which incorporates both qualitative and quantitative methods, will be employed. This approach was chosen for its ability to enable stakeholders and community members to actively participate in addressing the challenges faced by the community through a series of cycles, as shown in Figure 1. This protocol has been developed in accordance with recognised reporting guidelines to promote methodological rigour, transparency, and reproducibility. The intervention is described using the Template for Intervention Description and Replication (TIDieR) checklist to ensure comprehensive reporting of the intervention components and facilitate replication. The qualitative components of the study will be reported in accordance with the Consolidated Criteria for Reporting Qualitative Research (COREQ) checklist. Furthermore, the implementation process and implementation outcomes will be reported in accordance with the Standards for Reporting Implementation Studies (StaRI) statement, where applicable.

### 3.2 Study setting

The project will be conducted in Tshimbupfe Village, located in the Vhembe District of Limpopo Province, South Africa. Tshimbupfe Village was purposively selected as the implementation site based on findings from our baseline study, which showed lower utilisation of health facilities among men than in neighbouring villages, namely Dindela, Malonga, Manavhela, and Mavhulani. The selection of Tshimbupfe was therefore informed by evidence indicating a greater need for interventions to improve men’s health-seeking behaviour. These villages were identified during the baseline assessment solely to inform the selection of an appropriate implementation site and were not included for comparative analysis. The village has one health facility, which provides primary health care services to the community. The village is predominantly inhabited by Tshivhenda-speaking residents who maintain strong cultural traditions.

### 3.3 Study population and sampling

The target population in this project is the Department of Health Management, healthcare workers (Health Services Directorate) and facility teams (nurses, filing clerks), Community leaders, traditional healers and community members. Purposive sampling will be used to select stakeholders and community members who will serve on the committee. Total population sampling will be used to include all men aged 19 years and above who reside in rural settings. Although the exact number of study participants cannot be determined in advance, as the study employs total population sampling, it is anticipated that more than 400 men will participate in this implementation study. This estimate is based on the mobilisation efforts, involvement of community leaders and stakeholders, and the male population size in the study setting. Table 2 presents the anticipated sample size per category of stakeholders:

**Table 2:** Anticipated stakeholders sample size.

Sample	Size
Department of Health, PHC Directorate:	1
Facility managers:	1
Professional Nurses:	3
community leaders:	3
Traditional Healers:	3
Representatives of men living in the community:	5
Religious leaders:5	5
NGOs:	3
Males	400

### 3.4 Data instrument

Key informant interviews (KIIs) will be conducted with healthcare workers, community leaders, and traditional healers. Focus Group Discussions (FGDs) with purposively selected men who have participated in the implementation activities will also be held. The number of FGDs to be held will depend on data saturation. Community workshops will be held to adapt intervention strategies collaboratively. Pre- and post-evaluation self-administered questionnaires will be used before and after workshops. Data collection tools will be developed by the researchers and stakeholders as co-researchers. Developed data collection tools will be validated by consulting experts in the field on areas where researchers and the committee lack expertise. Moreover, routine health facility data from the local health facility will be collected continuously to establish the effectiveness of the strategies as soon as community dialogues, campaigns and workshops are completed. Collected data will focus on facility attendance, disease screening, attendance at preventive health check-ups, and improvement in retention of men in chronic medication. We will also liaise with local traditional healers to obtain reports on the number of men who were successfully referred to health facilities.

### 3.5 Strategies implementation process

Project Implementation will take place in different phases as follows:

#### 3.5.1 Phase 1: planning

The researcher will approach the Department of Health (DOH), the community leaders and traditional healers and request a meeting in the form of a workshop, as they are key stakeholders in the implementation of the strategies. A working committee will be established, and the proposed strategies to promote men’s health-seeking behaviour will be presented to familiarise committee members with the strategies. Stakeholders as co-researchers will be allowed to give input on the proposed strategies. PAR suggests that the researcher and stakeholders, as co-researchers, work together in identifying the problem and intervention; however, in this project, the identification of the problem and intervention will be skipped, as prior research has already identified the problem and provided suggested interventions. The identified problem was poor men’s health-seeking behaviour, and factors contributing to it were thoroughly studied before the development of the strategies. The identified factors included self-medication, Fear of knowing one’s own health status and disease screening, use of traditional healing services, masculinity beliefs, and stigma (14). Once the strategies are presented, a discussion on adopting the strategies will commence. A decision on which strategies will be implemented first and how effectiveness will be measured will be made collaboratively with the stakeholders. To encourage continuous stakeholder participation, the implementation committee, of which stakeholders are part, will oversee the rollout of the strategies, monitor progress, identify implementation challenges, and recommend modifications where necessary. Regular stakeholder meetings and reflection sessions will be conducted throughout the implementation period to review progress, discuss emerging issues, and collectively make decisions regarding adaptations to the intervention. The researchers will also establish and maintain collaborative working relationships with stakeholders. The researchers and the committee will further collaboratively identify other activities in addition to those outlined in the strategies that can be carried out to improve men’s health-seeking behaviour.

#### 3.5.2 Phase 2: action

Strategies that have been selected as priorities in improving men’s health-seeking behaviour will be implemented. Actions to be carried out will depend on what the committee and the researchers have agreed on. Community members’ active participation will be encouraged. Community leaders and stakeholders will play key roles in the recruitment of men as beneficiaries of the project. To ensure that the project extends beyond a conventional awareness campaign, the intervention will concentrate on putting into practice several evidence-based tactics that address individual, community, and health system barriers to men’s health-seeking behaviour. The strategies will be introduced, coordinated, monitored, and assessed through workshops, community discussions, and stakeholder meetings. In order to maximise engagement among men, especially men who may not regularly access health care, a community mobilisation strategy will be undertaken in the community before and during the intervention. The research team will engage with traditional leaders, ward councillors, community health workers, traditional healers, religious leaders, local employers, community-based organisations, and men\'s social groups to identify and recruit men who are less likely to seek healthcare to participate in the project. The workshops, community dialogues, and awareness campaigns will be advertised using community meetings, churches, local radio stations, social media, posters, and word-of-mouth. Intervention activities will be held at community venues that are easy to access, and, where possible, in the evenings or weekends to make them more accessible to employed men. Transport support and refreshments will be offered when resources permit. Traditional healers and community health workers will also encourage men who do not often visit health facilities to take part in the dialogues and campaigns. If the target population’s participation is lower than expected, more mobilisation efforts will be conducted.

#### 3.5.3 Phase 3: reflection and evaluation

Reflection sessions will be held to reflect on what has worked and what has not worked. Reflection sessions will also be used to gather participants’ experiences of participating in the PAR. To evaluate whether the intervention was implemented successfully and achieved its intended outcomes, both qualitative and quantitative data will be used to measure the effectiveness of the strategies. The findings will also be used to refine the strategies. Pre- and post-intervention questionnaires will be administered to measure changes in men’s willingness. Routine facility data will be collected to track clinic attendance by men over time and participation in disease screening. Tables 3, 4 provide the evaluation matrix and the suggested timeline per study phase.

**Table 3:** Evaluation framework.

Evaluation question	Outcome	Indicator	Data source	Data collection method
Were the intervention strategies implemented as planned?	Fidelity of implementation	Number of planned activities completed; proportion of strategies implemented	Implementation records, facilitator checklists	Document review and observation
Did the intervention reach the intended population?	Reach	Number and percentage of eligible men participating in workshops, dialogues and campaigns	Attendance registers	Attendance records
Were stakeholders actively involved in implementation?	Stakeholder participation	Number of stakeholder meetings held; attendance rate; stakeholder satisfaction	Meeting minutes, attendance registers	Document review and interviews
Did the intervention improve men’s knowledge of health-seeking behaviour?	Improved knowledge	Mean knowledge score before and after intervention	Pre- and post-questionnaires	Self-administered questionnaires
Did participants develop more positive attitudes towards healthcare utilisation?	Improved attitudes	Mean attitude score	Pre- and post-questionnaires	Self-administered questionnaires
Did the intervention improve men’s willingness to use healthcare services?	Increased willingness	Percentage willing to seek healthcare and undergo disease screening	Pre- and post-questionnaires	Self-administered questionnaires
Did utilisation of health services increase following implementation?	Increased utilisation	Number of male clinic visits; disease screening uptake; preventive health check-ups	Health facility records	Routine health information system
Did referrals from traditional healers increase?	Improved linkage to care	Number of men referred by traditional healers to health facilities	Traditional healer records and clinic registers	Record review
Did the intervention improve retention among men with chronic conditions?	Improved retention	Number of men remaining on chronic treatment; reduction in loss to follow-up	Health facility records	Record review
Was the intervention acceptable to participants?	Acceptability	Participants’ perceptions of usefulness, relevance and satisfaction	FGDs	Semi-structured FGDs
Was the intervention feasible to implement?	Feasibility	Stakeholder perceptions of practicality, resources and barriers	Key informant interviews	Semi-structured interviews
What behavioural changes occurred following implementation?	Improved health-seeking behaviour	Reported clinic attendance, screening uptake, early healthcare seeking	FGDs and questionnaires	FGDs and questionnaires

**Table 4:** Suggested implementation timeline.

Phase	Proposed activities	Duration
Phase 1: planning	Stakeholder engagement, formation of the PAR committee, orientation meetings.	Months 2 months
Phase 2: action (Implementation)	Community dialogues, workshops, awareness campaigns, engagement of traditional leaders,	Months 4
Phase 3: reflection and monitoring	Review meetings, reflection sessions, identification of implementation challenges, strategy refinement, and fidelity monitoring.	Months 2 months

### 3.6 Implementation fidelity

Implementation fidelity will be assessed using measures of reach, attendance, delivery, adherence, participant responsiveness, and process indicators to evaluate whether strategies were implemented as intended. The number of eligible men taking part in intervention activities will be used to evaluate reach. Participation in workshops and community discussions will be recorded using attendance registers. To ascertain whether intervention activities and strategy components were carried out as intended, facilitator checklists will be used to evaluate delivery and adherence. Participant responsiveness will be assessed using post-workshop evaluation questionnaires. The number of stakeholder meetings held, community mobilisation initiatives carried out, referrals made, men taking part in disease screening, the number of men lost to follow-up and linked back to care, and healthcare services are all examples of process indicators. These measures will assist in gathering evidence on the effectiveness of the interventions.

### 3.7 Data analysis

Audio-recorded interviews and discussions will be transcribed verbatim and analysed thematically. Repeatedly read transcripts, identify codes, group similar codes into categories and develop themes and sub-themes. Quantitative data will be captured in Excel and cleaned to ensure data quality and later transferred into Statistical Package for the Social Sciences(SPSS) version 29.0. Both descriptive and inferential statistics will be used to analyse quantitative data, with the significance level of the tests set at 0.05 and the CI set at 95%. Binary logistic regression analysis will be performed to determine the predictors of men’s willingness to use health services. The McNemar Test will be used to compare men’s willingness to use healthcare services and screen for diseases pre- and post-workshops. A chi-square test will be performed to measure the association between men’s educational level, age and willingness to participate in disease screening.

## 4 Discussion

This project addresses critical public health concerns of men’s poor health-seeking behaviour. The project aims to measure the effectiveness of the developed strategies in addressing this critical public health concern. The effectiveness of the strategies could improve public health in communities where implementation will take place. Improved men’s health-seeking behaviour could help reduce the rates of infections, reduce mortality and improve life expectancy. Effective implementation of the strategies could assist with the early detection and treatment of disease. Men who engage in health-seeking behaviour are more likely to seek early diagnosis, initiate treatment, and effectively manage health conditions. Improved men’s health-seeking behaviour can help reduce the burden of late-stage disease management and reduce costs to the health system. Successful implementation of the strategies enhances public health programmes’ effectiveness, especially programmes such as HIV and TB, where men rarely participate in screening. Furthermore, this project will provide practical insights into which strategies work; this strengthens evidence-based decision-making and supports the design of targeted interventions that are culturally appropriate in rural areas.

This project employs PAR, which allows the active involvement of stakeholders and collaboration between researchers and stakeholders in addressing men’s poor health-seeking behaviour in the study area. Moreover, the study builds on prior research that identified contributing factors to men’s poor health-seeking behaviour and developed and validated 14 evidence-based strategies. The study also employs both qualitative and quantitative methods, which strengthen a rich understanding of behaviour change and measurement of perceptions and actual outcomes. However, the study has several limitations. First, it will be conducted in a single rural village, which limits generalisability. The study runs a risk of selection bias. This is because participation is voluntary, and men who consent to participate may be those who are already interested in health issues and more willing to engage with health services than those who do not participate. However, extensive community mobilisation will be conducted to minimise selection bias. Moreover, social desirability bias is likely to occur when participants give responses that they think and believe are socially acceptable rather than expressing their true beliefs and views. To address social desirability bias, the self-administered questionnaires will be completed anonymously. Participants will also be given assurance of confidentiality on their responses and will be encouraged to provide honest responses. The study employs a pre-test and post-test design without a comparison group. Observed changes in men’s health-seeking behaviour may not be attributable exclusively to the study interventions, as other ongoing public health campaigns taking place during the study period may also influence outcomes. The study does not include the conventional PAR problem-identification phase because the problem and strategies were identified in an earlier study, even though stakeholders will take part in implementation and evaluation activities. As a result, compared to a full PAR cycle, some stakeholders might be less involved in the intervention’s early development. The study relies on self-reported data, which are susceptible to recall bias. The study mainly assesses results right after implementation. As a result, it might not be able to ascertain whether long-term improvements in health-seeking behaviour are sustained.
